# Correlation between caloric test results and VHIT VOR gains in unilateral horizontal canal deficits: a cross-sectional study

**DOI:** 10.1097/MS9.0000000000000427

**Published:** 2023-04-07

**Authors:** Khadija El Bouhmadi, Myriam Loudghiri, Youssef Oukessou, Sami Rouadi, Redallah Abada, Mohamed Roubal, Mohamed Mahtar

**Affiliations:** Department of Otolaryngology, Head and Neck Surgery, Ibn Rochd University Hospital, Faculty of Medicine and Pharmacy, Hassan II University, Casablanca, Morocco

**Keywords:** caloric test, VHIT, VOR gain

## Abstract

The purpose of the study is to evaluate the correlation between caloric test results and video head impulse test (VHIT) vestibulo-ocular reflex (VOR) gains in unilateral horizontal canal deficits in order to define a possible threshold value above which caloric deficits should be associated with predictable low VHIT VOR gains. Caloric test and VHIT were realised in 105 patients presenting with symptoms of rotational vertigo occurring within the last 2 weeks. The authors defined the cutoff value for a caloric abnormality as more than 15% of canal deficit, which allowed us to divide our patients on groups based on the severity of their caloric asymmetry. Then, the authors performed the VHIT considering abnormal horizontal gain as less than 0.8 with catch-up saccades. The authors evaluated the prevalence of results dissociation between the two tests and the correlation between the caloric asymmetry and the horizontal VHIT VOR gains in each group according to the severity of canal deficit. The correlation was considered statistically significant if *P* less than 0.05 (Fisher’s exact test). The caloric test revealed a significant unilateral deficit in 50 patients (47.6%). The interval of deficit between 21 and 40% included 25 patients, 18 (72%) presenting with normal VHIT VOR gains versus 7. On the other hand, for the 12 patients in the intervals of 81–100% of deficit, the VHIT VOR gain was highly abnormal in all cases. In comparison with the normal caloric test group, a correlation between each interval of caloric deficits and VHIT VOR gains has been assessed. This correlation was significant in the interval of 41–60% (*P*=0.04 <0,05) and in the interval of 81–99% next to patients with a total deficit of 100% (*P*=0.006 <0.05 for each). It appears that simultaneous affection of high vestibular frequencies evaluated on VHIT may be more likely and predictable above a minimal threshold of 40% caloric asymmetry, with better discrimination between normal and abnormal VHIT above 80%. Thus, they are two complementary tests to use as a couple rather than a replacement one for the other.

## Introduction

Highlights

- Caloric test and video head impulse test (VHIT) show different responses of the vestibulo-ocular reflex since they stimulate different frequencies.
- Simultaneous affection of high vestibular frequencies evaluated on VHIT may be more predictable above a minimal threshold of 40% caloric asymmetry.
- VHIT and caloric data provide unique information on the functional integrity of the horizontal semicircular canals at different points on the frequency spectrum.
- They are two complementary tests rather than a replacement one for the other.

The details of Robert Barany’s caloric test were first published in a journal in 1906, based on his clinical observation of nystagmus after external ear canal irrigation in order to remove cerumen, whose direction depends on the water temperature. Since his work on the caloric reaction had been awarded the Nobel Prize in Medicine in July 1916, the caloric test remains the gold standard to investigate peripheral vestibular dysfunction^1^.

The Head Impulse Test described by Halmagyi and Curthoys in 1988 enables the individual and rapid screening for each of the six semicircular canals (SCC) via fast head movements in the three planes. However, its interpretation suffered from the very short duration of the vestibulo-ocular reflex (VOR) making it hardly interpretable, mostly for vertical canals. The Video Head Impulse Test (VHIT) is the computerized version of the Head Impulse Test, based on a high-resolution video camera connected to a computer for automatic head and eye movements’ analysis, identifying overt and covert saccades, and calculating the VOR gains^2^,^3^.

These two tests activate different frequency ranges, through different stimulation pathways, aiming different receptors. Various studies showed that both tests may not always identify vestibular hypofunction, and their results might even be contradictory. But they are both contributory, since a comprehensive vestibular assessment requires the ability to measure the global function of its six SCC and four otolith organs in response of all the frequency spectrum stimulations.

## Aim of the study

The aims of our study is to evaluate the relationship between Caloric Test results and VHIT VOR gains in unilateral horizontal canal deficits, in order to demonstrate when low vestibular frequencies affections are correlated to abnormalities in the high frequencies too and to define a possible threshold value above which caloric deficits should be correlated to predictable low VOR gains.

## Patients and method

A total of 105 patients presenting with vertigo were enrolled in this study (sample size calculated to meet 95% of CI with 5% of margin of error for a population proportion of 5% according to the vestibular vertigo 12-month prevalence reported in the literature^4^). They were suitable for inclusion to the study if they were presenting with rotational vertigo occurring within the last 2 weeks (first time incident or a new episode of recurrent vertigo). After history inquiring, otology and audiology examination, clinical examination under videonystagmoscopy, they fulfilled the vestibular instrumental tests: caloric irrigation followed by VHIT.

All patients with focal central neurological symptoms (such as sensory loss, difficulty speaking, and severe postural instability) or central ocular motor abnormalities or central nystagmus at the clinical examination were excluded from the study. Patients were also excluded if they were unable to perform both vestibular tests (caloric irrigation and VHIT).

We defined the cutoff value for a caloric abnormality as more than 15% of canal deficit, which allowed us to divide our patients on groups based on the severity of their caloric asymmetry. Then, we performed the VHIT considering abnormal horizontal gain as less than 0.8 with corrective catch-up saccades.

We evaluated the prevalence of results dissociation between the two tests and the correlation between the caloric asymmetry and the horizontal VHIT VOR gains in each group according to the severity of the canal deficit. The correlation was considered statistically significant if *P* less than 0.05 (Fisher’s exact test).

The work has been approved by the ethical committee of our department. All the patients gave their consent for the examination leading to the results of this study. The paper was written meeting the strengthening the reporting of cohort, cross-sectional and case-control studies in surgery (STROCSS) 2021 Criteria and was registered under the number 8673 on the Research Registry^5^ https://www.researchregistry.com/browse-the-registry#home/registrationdetails/63ea1dde446f070011301b69/.

## Results

Among the 105 patients, there was a female predominance (23 M/82 F) with a sex ratio at 0.28. The median age was 48.16 years old [13–70 years]. 45 patients (42.85%) presented with first time vertigo attack while 60 patients (57.14%) reported recurrent vertigo. Also, audiological symptoms such as hearing loss and tinnitus were found in 38 patients (36.19%).

**Figure 1:**
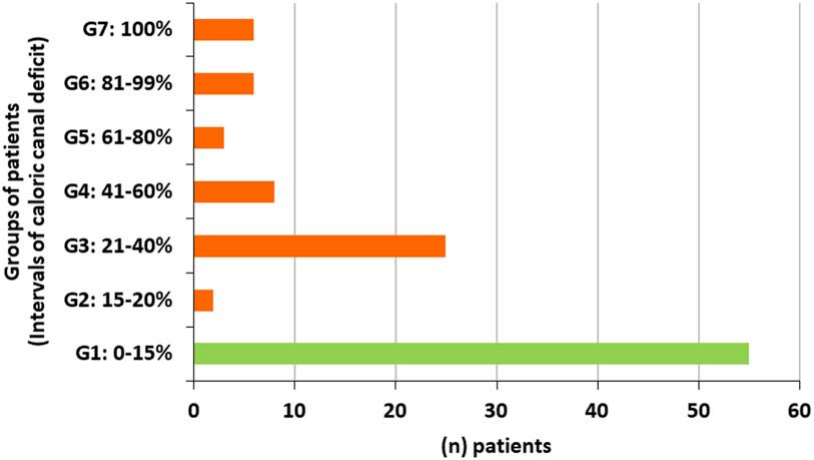
Distribution on groups based on the severity of caloric canal deficit.

According to their caloric test results, we separated our patients in seven groups. The group one counted 55 patients, having normal caloric test. Unilateral horizontal canal deficit was observed in 50 patients distributed in groups of increasing deficit values as follow (Fig. 1).

Mean values of canal deficits were calculated for each group (Table 1).

**Table 1:** Mean values of canal deficits for each group

G1	G2	G3	G4	G5	G6	G7
6.14%	19%	29.28%	50.12%	67%	94%	100%

Then, horizontal VHIT was realised in each group, notified as normal or abnormal according to the VOR gains (Table 2).

**Table 2:** Results of horizontal VHIT for each group

	Caloric test deficit (%)	Normal VHIT VOR gain	Low VHIT VOR gain
Group 1	0–15	41	14
Group 2	15–20	2	0
Group 3	21–40	18	7
Group 4	41–60	3	5
Group 5	61–80	1	2
Group 6	81–99	0	6
Group 7	100	0	6

* Prevalence of dissociation in the results between caloric tests and horizontal VHIT:

Most of the patients (67=63.80%) showed consistent results in the caloric test and horizontal VHIT: being normal on both tests in 41 patients (39.04%) and abnormal on both tests in 26 (24.76%). While dissociated results between the two tests were found in 38 patients (36.19%): an abnormal caloric test but a normal VHIT in 24 (22.85%) and a normal caloric test but an abnormal VHIT in 14 (13.33%) (Table 3).

**Table 3:** Prevalence of dissociation in the results between caloric tests and horizontal VHIT

	Normal VHIT	Abnormal VHIT
Normal caloric test (G1)	41 (39.04%)	14 (13.33%)
Abnormal caloric test	24 (22.85%)	26 (24.76%)

* Correlation between caloric test results and horizontal VHIT VOR gains according to the severity of canal deficit:

More specifically, we studied the correlation of these two tests in each group.

The interval of deficit between 21 and 40% (Group 4) included 25 patients, 18 (72%) presenting with normal VHIT VOR gain versus 7. On the other hand, for the 12 patients in the intervals of 81–100% of deficit (Groups 6 and 7), the VHIT VOR gain was highly abnormal in all cases.

So, in comparison with the 55 patients with a normal caloric test (Group 1), whose 14 had low VOR gains, a correlation between each interval of caloric deficits and VHIT VOR gains has been assessed.

This correlation was significant in the groups 4, 6, and 7 with, respectively, *P*=0.04 and *P*=0.006 for both. Thus, our study showed that a minimal threshold of 40% caloric asymmetry is required to optimize discrimination between normal and abnormal VHIT, with better discrimination above 80%. The absence of a significant correlation in the Group 5 with an interval between 61–80% may be explained by the low number of patients (Table 4).

**Table 4:** Correlation between caloric test results and horizontal VHIT VOR gains according to the severity of canal deficit

	Number of patients	Caloric test deficit (%)	Normal VHIT VOR gain	Low VHIT VOR gain	*P*
Group 1	55	0–15	41	14	
Group 2	2	15–20	2	0	1
Group 3	25	21–40	18	7	0.79
Group 4	8	41–60	3	5	0.04
Group 5	3	61–80	1	2	0.18
Group 6	6	81–99	0	6	0.006
Group 7	6	100	0	6	0.006

Bold values denotes *P* is the result of Fisher’s exact test calculation, considering the correlation significant if <0.05

## Discussion

### Caloric Test / VHIT: Different frequency range, different stimulation pathways, different receptors

**Figure 2:**
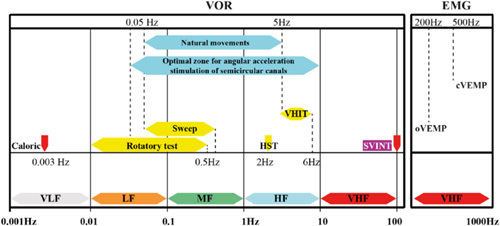
Graph summarizing the complementarity of vestibular tests in the currently known frequency spectrum of the vestibular system^6^.

Caloric test and VHIT aim different frequency ranges. VHIT evaluates high frequencies above 5 Hz using rapid and short head impulses, whereas caloric irrigation activates lower frequency bands at 0.003 Hz^6^ (Fig. 2).

Also, the differences between the VHIT and the caloric test lie on the alterations documented by the VOR. Indeed, caloric test relies on a slow and indirect type 2 neuron pathway, inducing an endolymphatic flow via a temperature gradient, independent of gravity and involving storage velocity. While VHIT follows a fast and direct type 1 neuron pathway, generating a physiologic endolymphatic flow via rapid head impulses with transmission from the SCC directly to the ocular effector muscles without modulation^7^.

### Correlation between Caloric test results / VHIT VOR gains

Beyond the differences, some authors reported an effective linear correlation between these two tests, as in our study, with consistent results in 63.80% of the cases. In a study on 60 patients with unilateral vestibular disease, there was a statistically significant negative correlation between the VOR gain of the affected ears and the caloric deficit. (*r*=−0.513, *P*<0.0001). Also, the mean value of caloric deficit (65.5%) was higher in the overt saccade group with a statistically significant difference (*P*=0.006)^8^.

More precisely, this correlation seemed to be mainly linked to the importance of caloric asymmetry, the disease stage, and the test itself. Just like our findings, some studies have observed that, even with dissociated results, there seems to be a cutoff value of caloric asymmetry, near 40%, above which caloric deficits should be correlated to predictable low VOR gains. But this threshold was interpreted as a lack of sensitivity of the VHIT since the deficit already significant in the caloric test at 22–25% becomes significant in the VHIT until it reaches 40–60%^9^,^10^.

On the other hand, as for 36.19% of our patients, other studies reported total dissociation of the caloric test and VHIT results, even for more than 40% of caloric canal paresis (*P*=0.1779). They concluded then, that lateral VHIT VOR gains do not predict the degree of lateral semicircular deficit^7^. Also, dissociation in test findings can be related to changes in measurable VHIT results as compensation progresses, whereas the caloric asymmetry remains more stable, since nerves encoding high frequency vestibular stimuli recover at different rates than the lower frequency responses assessed by calorics^11^.

### Comparison Caloric test / VHIT

Above the results, to compare the two tests, VHIT offers a high specificity (90%), screening of the six SCC individually through natural frequency movements, which is very relevant in acute dizziness to differentiate between central and peripheral lesions and providing an insight into the state of the patient’s central compensation^11^. While the caloric still test the vestibule on nonphysiologic frequencies. In addition to the fact of being time-consuming and unpleasant, it provides a qualitative and quantitative evaluation of vestibular function by comparing the two sides.

There is presently a debate in the scientific community as to whether the VHIT will replace the bithermal caloric test. But for now, their results may be diverse, but they complete each other since they both describe the tonotopy of the crista ampullaris depending on the stimulation frequency^7^.

However, our expectation on the contribution of the test on the diagnosis should be directed by the clinical context. The Caloric seems more adequate for screening patients with chronic complaints. While vestibular neuritis may produce abnormal VHIT but normal caloric test during the acute phase. Also, abnormal caloric response but normal VHIT may be observed in Meniere’s disease^7^,^12^,^13^.

### Strengths and limitations of the study

Our study enrolled a representative sample of patients with vertigo, all vestibular affections combined, which can generalize the research findings. Also, the assessment of the correlation between their caloric test results and VHIT VOR gains provided a precise numerical threshold that would improve the test’s interpretation.

However, the variability of the results over time according to the pathology evolution and the level of compensation remains a limit to our study. Also, evaluating this correlation in groups of patients with the same vestibular disease would reinforce the data on the expected results of vestibular testing in each pathological indication.

## Conclusion

Caloric test and VHIT show different responses of the VOR since they stimulate different frequencies. However, it appears that simultaneous affection of high vestibular frequencies evaluated on VHIT may be more likely and predictable above a minimal threshold of 40% caloric asymmetry, with better discrimination between normal and abnormal VHIT above 80%.

VHIT and caloric data provide unique and complementary information regarding the functional integrity of the horizontal SCC at different points on the frequency spectrum. Indeed, they are two complementary tests to use as a couple rather than a replacement one for the other.

## Ethical approval

This work has been approved by the ethical committee of our department. All the patients gave their consent for the surgery and for the follow up leading to this study.

## Consent

All the patients gave their consent for the surgery and for the follow up leading to this study. Written informed consent was obtained from all the patients for publication of their results.

## Sources of funding

None.

## Conflicts of interest disclosure

The authors declare that they have no financial conflict of interest with regard to the content of this report.

## Provenance and peer review

Not commissioned, externally peer-reviewed.
